# Lack of evidence for conserved parasegmental grooves in arthropods

**DOI:** 10.1007/s00427-022-00684-5

**Published:** 2022-01-17

**Authors:** Ralf Janssen, Natascha Turetzek, Matthias Pechmann

**Affiliations:** 1 Department of Earth Sciences, Uppsala University, Villavägen 16, 75236 Palaeobiology, Sweden; 2 Evolutionary Ecology, Faculty of Biology, Ludwig-Maximilians Universität München, Grosshaderner Strasse 2, 82152 Biozentrum, Germany; 3 Institute for Zoology, Department of Developmental Biology, University of Cologne, Zuelpicher Str. 47b, 50674 Biocenter, Germany

**Keywords:** Segment-polarity, Engrailed, Segmentation, Parasegment, Neo-functionalization, Arthropod development

## Abstract

In the arthropod model species *Drosophila melanogaster*, a dipteran fly, segmentation of the anterior–posterior body axis is under control of a hierarchic gene cascade. Segmental boundaries that form morphological grooves are established posteriorly within the segmental expression domain of the segment-polarity gene (SPG) *engrailed* (*en*). More important for the development of the fly, however, are the parasegmental boundaries that are established at the interface of *en* expressing cells and anteriorly adjacent *wingless* (*wg*) expressing cells. In *Drosophila*, both segmental and transient parasegmental grooves form. The latter are positioned anterior to the expression of *en*. Although the function of the SPGs in establishing and maintaining segmental and parasegmental boundaries is highly conserved among arthropods, parasegmental *grooves* have only been reported for *Drosophila*, and a spider (*Cupiennius salei*). Here, we present new data on *en* expression, and re-evaluate published data, from four distantly related spiders, including *Cupiennius*, and a distantly related chelicerate, the harvestman *Phalangium opilio*. Gene expression analysis of *en* genes in these animals does not corroborate the presence of parasegmental grooves. Consequently, our data question the general presence of parasegmental grooves in arthropods.

**Supplementary Information:**

The online version contains supplementary material available at 10.1007/s00427-022-00684-5.

## Introduction

In the fruit fly *Drosophila melanogaster*, segmentation of the anterior–posterior (AP) body axis proceeds through the action of the so-called segmentation genes, i.e., gap, pair-rule, and segment-polarity genes. At the end of this cascade, pair-rule genes (PRGs) such as *even-skipped* (*eve*) and *fushi-tarazu* (*ftz*) ensure that segment-polarity genes (SPGs) such as *engrailed* (*en*) and *wingless* (*wg*) are expressed in segmentally repeated stripes (reviewed in Sanson 2001). Expression of *en* and *wg* is initiated in adjacent regions of every segment, *wg* anteriorly abutting the domain of *en*. After the initiation of this pattern by the PRGs, a positive auto-regulatory loop between *en* and *wg* expressing cells maintains their expression. The border between *en* and *wg* expressing cells is the parasegmental border, which acts as the primary organization center of the segment and functions as a clonal boundary (e.g., Vincent and O’Farrell 1992). The segmental boundaries that are represented by grooves form posterior to the expression of *en* (or in the most posterior *en* expressing cells). These are the morphological boundaries that are later seen in the larva and also in the adult fly. At least in *Drosophila*, morphological grooves also form at the parasegmental boundary, i.e., at the interface between *en* and *wg* expressing cells (anterior to *en*), but the function of these grooves is not known (e.g. Larsen et al. 2008). Interestingly, parasegmental grooves have also been reported in a distantly related arthropod, the spider *Cupiennius salei* (Damen 2002). A later study in the brine shrimp *Artemia franciscana* suggested that parasegmental groove may also (although less obviously) form in crustaceans (Prpic 2008). These data combined led to the opinion that parasegmental grooves indeed represent a conserved feature of arthropod development (e.g. Deutsch 2004; Mellenthin et al. 2006; Chipman 2010; Franke and Mayer 2014). Parasegments marked by the highly conserved expression of the SPGs in arthropods appear to be conserved among arthropods, but the occurrence of parasegmental grooves has indeed only been reported for the above-mentioned species. Some authors explicitly mention that they have never observed parasegmental grooves in their model organisms (i.e., Chipman et al. 2004; Brena et al. 2006). Published data on SPG expression in other spiders than *Cupiennius* also do not mention parasegmental grooves, although these studies indeed are not focusing on this topic (e.g., Turetzek and Prpic 2016; Pechmann 2020).

We therefore decided to carefully analyze the expression of the SPG *en* with respect to the presence or absence of parasegmental grooves in a number of spider species that cover most main branches of spiders, true spiders such as *Parasteatoda tepidariorum* (Entelegynae) and *Pholcus phalangioides* (Haplogynae) and the tarantula *Acanthoscurria geniculata* (Mygalomorphae). In neither of these species, we found grooves forming anterior to the expression of *en* showing that clearly visible parasegmental grooves as described by Damen (2002) for the spider *Cupiennius* may not form at all in these investigated species. We then re-investigated the expression of *en* in *Cupiennius*, a species that represents a different subgroup of Entelegynae (i.e., the RTA clade (e.g., Garrison et al. 2016)) to which *Parasteatoda* does not belong, and for which parasegmental grooves have indeed been reported (Damen 2002)). Even in this species, however, we were unable to identify parasegmental grooves. This suggests that the earlier report by Damen (2002) must have interpreted the data incorrectly.

We also found that all spiders and closely related groups of chelicerates that belong to Arachnopulmonata (e.g., spiders, scorpions, whip scorpions) all possess two paralogs (ohnologs) of *en*. The second paralog (*en2*) likely evolved new functions in this group of chelicerates after a whole genome duplication (WGD) that took place in the lineage leading to arachnopulmonate chelicerates (Schwager et al. 2017).

## Methods

Sequence information of *en* genes have been identified in a sequenced genome (*Parasteatoda* (Schwager et al. 2017)) and sequenced embryonic transcriptomes (*Cupiennius* (Samadi et al. 2015), *Pholcus* (Janssen et al. 2015), *Acanthoscurria* (Pechmann 2020), *Phalangium* (Sharma et al. 2012), *Marpissa muscosa* (Harper et al. 2020), *Charinus acosta* (Harper et al. 2020), and *Euphrynichus bacillifer* (Harper et al. 2020). Potential orthologs were identified performing reciprocal tBLASTn searches against the single *en* gene of the onychophoran *Euperipatoides kanangrensis* (Eriksson et al. 2009). Phylogenetic analysis of *en* genes was done as described previously (Panara et al. 2019). The protein alignment and the nexus file are provided as Supplementary Files 1 and 2; gene identifiers of all genes used in this paper are summarized in the Supplementary File 3. Fragments of the genes were amplified using RT-PCR with gene-specific primers on cDNA that was reverse transcribed from total RNA isolated from embryos of different developmental stages; sequences of all used primers are summarized in Supplementary File 3. All fragments were cloned into the pCR-II Vector (Invitrogen) or the pJet1.2 CloneJet PCR cloning kit (Fisher Scientific) and sequenced on an ABI3730XL analyzer using Big Dye dye-terminators. In situ hybridization was performed as described in Janssen et al. (2018, supplement) and Panara et al. (2019) (FastRed staining for confocal microscopy). Cell nuclei staining was done by incubation of the embryos in 1:10,000 Sybr-green or 1 µg/ml 4–6-diamidino-2-phenylindole (DAPI) in phosphate-buffered saline with 0.1% Tween-20 (PBST) for 20–30 min. Stained embryos were investigated and photographed under a Leica dissection microscope that was equipped with a Leica DC100 digital camera. For confocal microscopy, we used a Leica SP8 inverted microscope. Embryos used for confocal microscopy were mounted in 1% low melting agarose. Confocal images were processed in Fiji/ImageJ (Schindelin et al. 2012). The image processing software Adobe Photoshop CC2018 was used for linear corrections of brightness and contrast.

## Results and discussion

### Spiders and other arachnopulmonate chelicerates retained two *engrailed *paralogs after whole genome duplication (WGD)

**Fig. 1:**
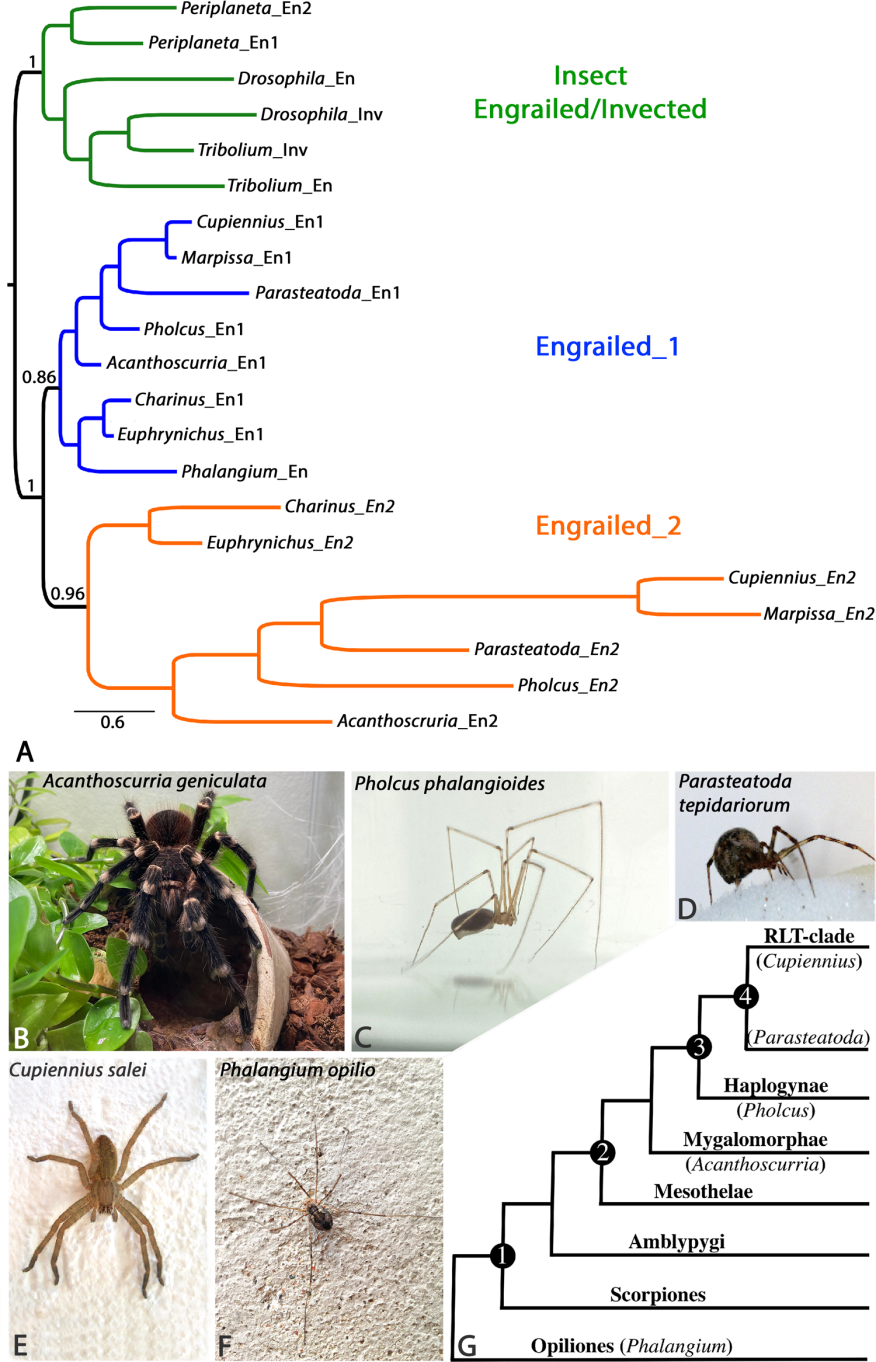
Phylogenetic tree of chelicerate *en1* and *en2* genes, research animals and their phylogenetic relationship. (A) Bayesian analysis of Engrailed (En) protein sequences in arachnopulmonate chelicerates (*Pholcus phalangioides*; *Cupiennius salei*; *Acanthoscurria geniculata*; *Marpissa muscosa*, *Parasteatoda tepidariorum*; *Charinus acosta*; and *Euphrynichus bacillifer*), the harvestman *Phalangium opilio*, and insects (*Drosophila melanogaster*; *Tribolium castaneum;* and *Periplaneta americana*). The analysis is based on the complete protein sequences. The scale bar indicates 0.6 amino acid substitutions per site. The chelicerate engrailed-1 sequences are depicted in blue. Arachnopulmonate-specific engrailed-2 sequences and depicted in orange. Insect engrailed and invected sequences are shown in green. (B–F) The main research organisms used in this paper. (B) The tarantula *Acanthoscurria*. Shown is an adult male. (C) An adult female of the cellar spider *Pholcus*. (D) Adult female of the common house spider *Parasteatoda*. (E) Juvenile of the American Wandering spider *Cupiennius*. (F) Adult female of the harvestman *Phalangium*. (G) Cladogram showing the phylogenetic relationship of chelicerates used in this study: 1, Arachnopulmonata; 2, spiders; 3, true spiders; 4, Entelegyne spiders

In the spiders, *Parasteatoda*, *Pholcus*, and *Acanthoscurria*, so far only one *en* gene was isolated in previous studies (Pechmann et al. 2009; Turetzek and Prpic 2016; Pechmann 2020). However, in *Cupiennius*, two paralogs were described (*engrailed-1* (*en1*) and *engrailed-2* (*en2*) (Damen 2002)). We identified an *en2* paralog in all investigated spiders, as well as in whip scorpions and a scorpion suggesting that all (or at least most) arachnopulmonate species may have retained two copies (ohnologs) of *en* after WGD (Schwager et al. 2017; Harper et al. 2020). Our phylogenetic analysis supports this as it shows monophyletic groups of *en* genes representing *en1* and *en2* respectively (Fig. 1). The most striking difference between the protein sequences of En1 (= En) and En2 is the lack of the C-terminal aspartic acid (D)- and glutamic acid (E)-rich domain that is usually present in En proteins, and a serine (S)-rich domain present in spider, whip-scorpion, mite, and harvestman En1 (En) proteins (Supplementary File 4).

### Expression of *engrailed *genes reveals the absence of clearly-visible parasegmental grooves in spider development

In his influential paper, Damen (2002) described for the first time the expression of the SPG *en* (two paralogs) in a spider. Based on the conserved expression patterns of this gene, he concluded that the parasegmental boundaries that are set by the same genes in *Drosophila* and other arthropods are conserved in spiders (Damen 2002, and references therein). Additionally, Damen (2002) reported on the presence of transient morphologically visible parasegmental boundaries (grooves) that allegedly form anterior to the segmental expression of *en*. Based on his finding, and the fact that parasegmental grooves exist in *Drosophila* (e.g., Martinez-Arias and Lawrence 1985; Larsen et al. 2008), he argued that parasegmental grooves are conserved and that they are thus representing an ancestral feature of arthropod development.

Although we fully agree with the idea of conserved parasegmental boundaries in spiders and indeed arthropods in general, we question the presence of transient parasegmental *grooves* in spiders. This is because expression of *en* genes in spiders other than *Cupiennius* has been studied, but neither of the corresponding papers described parasegmental grooves (e.g., Pechmann et al. 2009; Akiyama-Oda and Oda 2010; Turetzek and Prpic 2016; Pechmann 2020). This, however, may be due to the fact that these papers did not specifically address groove formation during segmentation. Additionally, numerous studies that provide comprehensive data on SPG expression (including *en*) in a large variety of different arthropod species did not report on the presence of parasegmental grooves either (e.g., Brown et al. 1994; Peterson et al. 1998; Marie and Bacon 2000; Hughes and Kaufman 2002; Kettle et al. 2003; Janssen et al. 2004; Chipman et al. 2004; Alwes and Scholtz 2006; O'Donnell and Jockusch 2010; Janssen 2012; Nakagaki et al. 2015; Lim and Choe 2020).

**Fig. 2:**
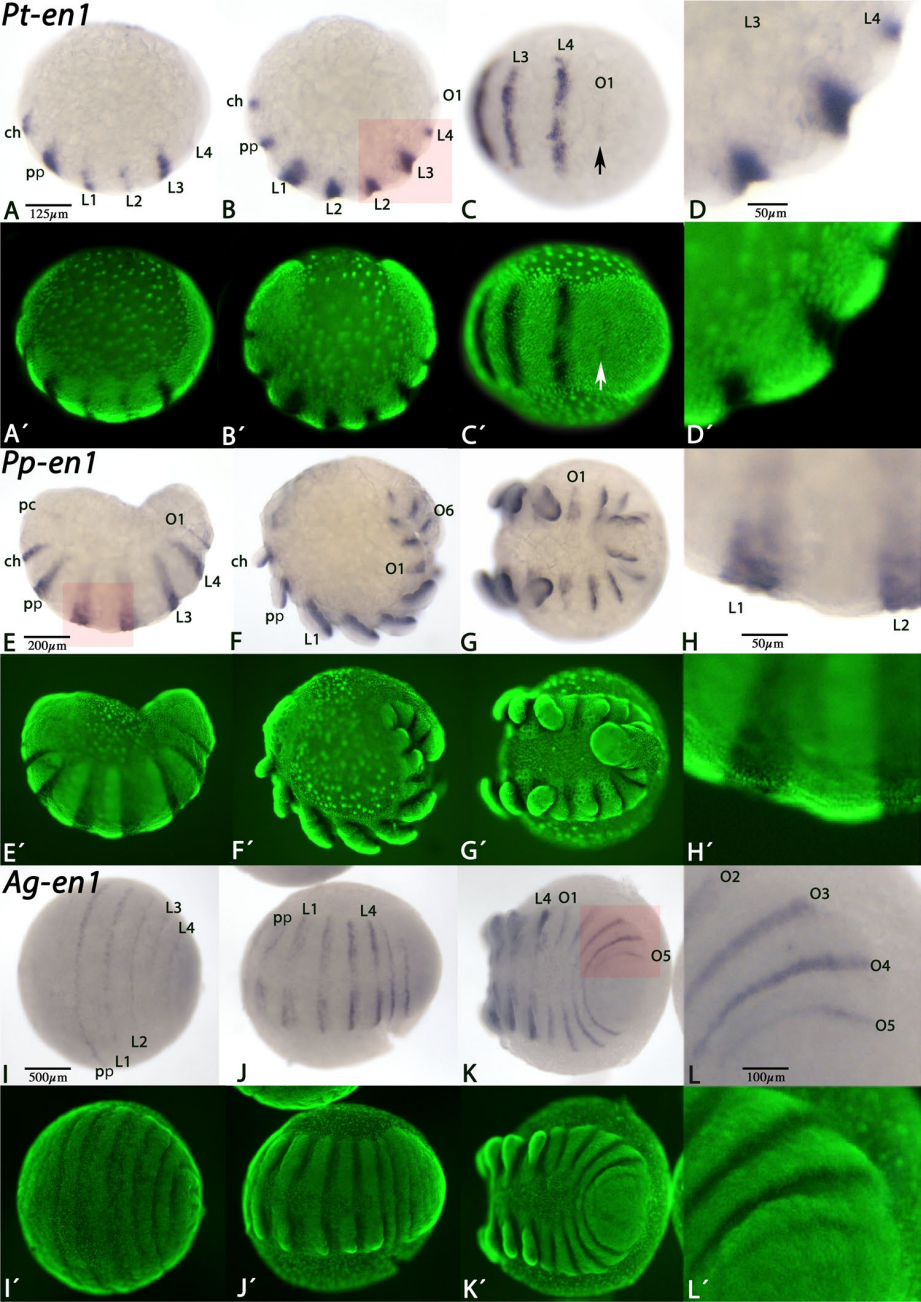
Expression of spider *en1*. In all panels, anterior is to the left. Panels A, B, E, F, and H show lateral views. Other panels show ventral views. Panels A´-L´ represent Sybr-green staining of the embryos shown in panels A–L. Red squares in panels C, E, and K shown regions that are enlarged in panels D, H, and L, respectively. The arrow in panels C/C´ points to earliest expression of *en-1* in the newly forming segment; there is no morphological groove anterior to this domain of expression. Note the general absence of morphologically visible grooves anterior to the expression of *en-1*. Abbreviations: ch, chelicera (cheliceral segment); L, leg-bearing segment; O, opisthosomal segment; pp, pedipalp (pedipalpal segment)

**Fig. 3:**
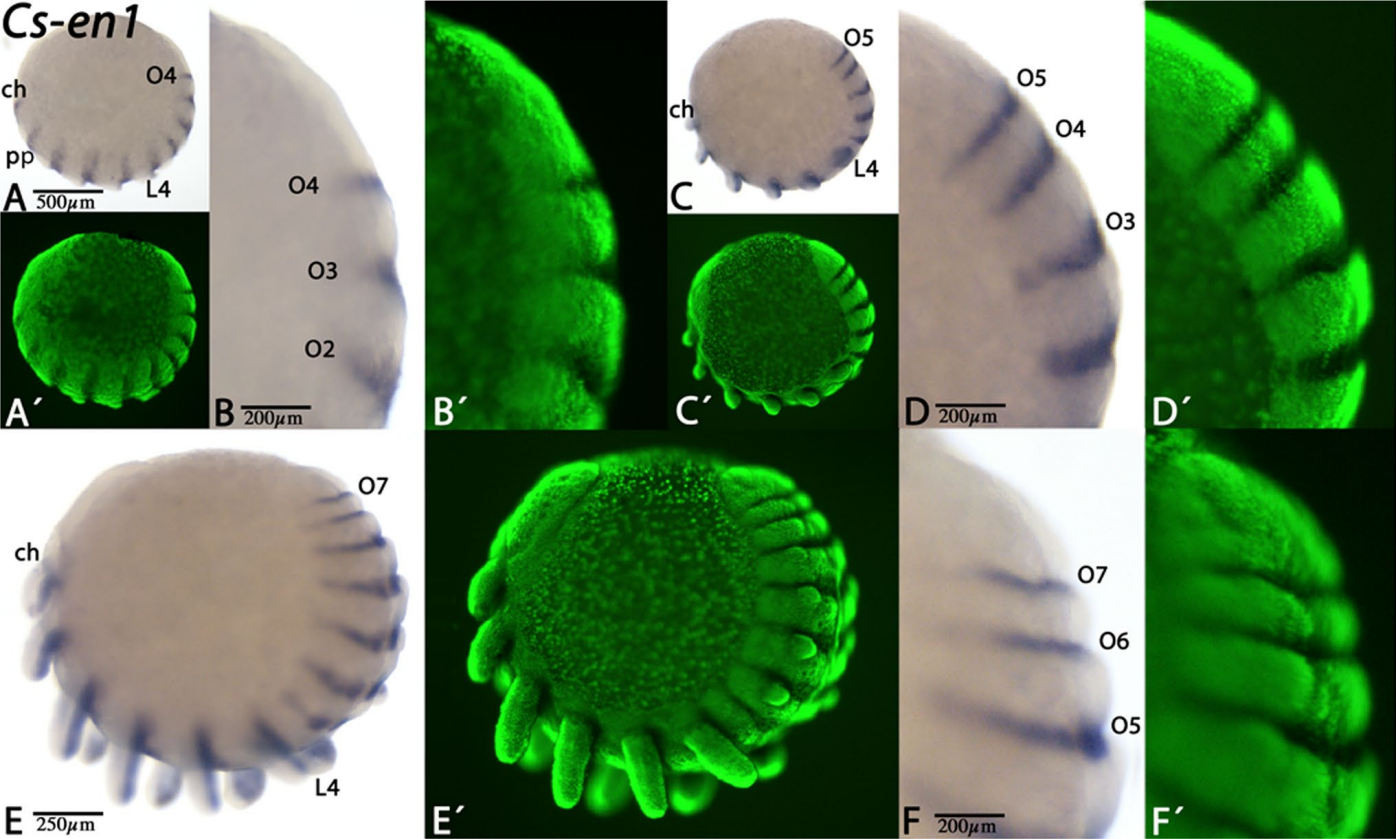
Expression of *Cupiennius en1*. In all panels, anterior is to the left, except for panels B, D, and F (anterior facing downwards). All panels represent lateral views. Panels A´–F´ represent Sybr-green staining of the embryos shown in panels A–F. Note the absence of morphologically visible grooves anterior to the expression of *en-1*. Abbreviations as in Fig. 2

Therefore, we first carefully analyzed the expression pattern of *en1* in *Parasteatoda* (Entelegynae), *Pholcus* (Haplogynae), and *Acanthoscurria* (Mygalomorphae) with respect to the potential formation of parasegmental grooves that, if present, would form anterior to the segmental expression of *en1*. However, in neither of the investigated species, *en1* is ever expressed posterior adjacent to any visible transverse morphological groove, but instead grooves are always associated with the most posterior segmental expression of *en1* (Fig. 2), suggesting that these grooves indeed represent the “normal” segmental grooves that are present in all arthropods. Since we did not find any evidence for the formation of parasegmental grooves in any of these spiders, we then carefully re-investigated the expression of *en1* in *Cupiennius*. To our surprise, we found that even in this species expression of *en1* is clearly aligned with the formation of the segmental grooves (Fig. 3). Our data on the expression of *Cupiennius en1* conflicts with the provided data by Damen (2002). However, together with our data on *en1* expression in various spider species, we conclude that the grooves reported in *Cupiennius* represent segmental borders, and not parasegmental borders.

**Fig. 4:**
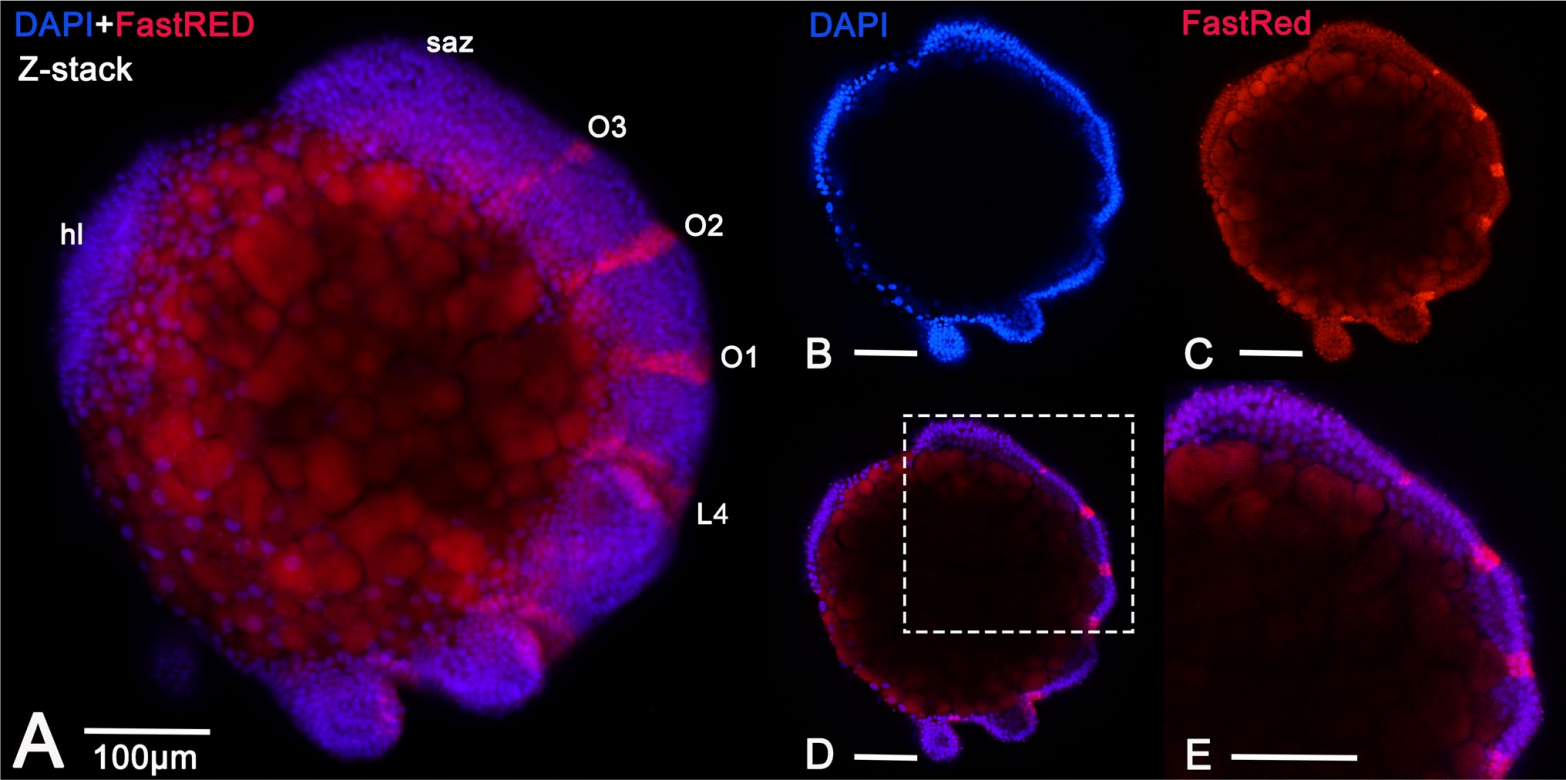
Confocal imagining of *Parasteatoda engrailed-1* expression. In all panels, anterior is to the left, lateral views. Panel A shows an overlay of the DAPI signal (blue) and FastRed staining in a partial scan (Z-stack) through a *Pt-en1* stained embryo. Panels B and C show one focal plane detecting DAPI and FastRed, respectively. Panel D shows the overlay of panels B and C. Panel E shows a magnification of the posterior part of the embryo as shown in panel D (dashed white box). All scale bars represent 100 µm. Abbreviations as in Fig. 2

In order to get better insight into the morphology of the germ band, we also investigated its morphology before and around the onset of *en1* expression in one spider, *Parasteatoda*, by means of confocal microscopy. We combined the fluorescent signal of the expression of *en1* (FastRed, Supplementary Fig. 1) with the nuclear dye DAPI (Fig. 4). Confocal microscopy offers a much higher resolution and allows to scan through all planes of a given embryo. We scanned embryos in slices of 1 µm and could not detect any sign of a groove anterior to *en1* (FastRed in-situ hybridization signal; see Supplementary Fig. 1). Neither the position of nuclei (DAPI signal) nor the surface of the cells (visualized by the background signal of the FastRed stain) indicates the presence of grooves (Fig. 4).

### Evolutionary significance

Engrailed and other SPGs are responsible for maintaining the conserved parasegmental borders in *Drosophila* and other arthropods (reviewed in Sanson 2001). Interestingly, the same genes appear to be responsible for segment border formation in annelids as shown for the nereid *Platynereis dumerilii*, thereby suggesting that the last common ancestor of protostomes was already segmented in at least some form and that the SPG-network was part of its segmentation process (Dray et al. 2010). In the annelid, however, segmental grooves (morphological segmental boundaries) form at the interface of *en*- and *wg/Wnt1*-expressing cells—where also the parasegmental boundaries of arthropods form. Conserved parasegmental grooves in arthropods would then be homologous to the segmental grooves of annelids. By showing that the parasegmental *grooves* are not a conserved ancestral feature of arthropod segmentation, our data weaken the idea that segmentation and groove formation in arthropods and annelids are conserved and thus question the idea of a segmental protostomian ancestor.

### Expression of *en2 *reveals aspects of sub- and neo-functionalization after WGD

Arachnopulmonate chelicerates retained two copies of *engrailed* after WGD. Other chelicerates such as harvestmen and mites, however, ancestrally only possess one *en* gene (*en1*) (Fig. 1). In all chelicerates, *en1* is expressed in the form of transverse segmental stripes suggesting that its function as a SPG is conserved (Figs. 2 and 3) (Telford and Thomas 1998; Damen 2002; Sharma et al. 2012; Turetzek and Prpic 2016; Sharma 2017; Pechmann 2020). Segmental grooves form in the posterior sector of this domain, as it is also the case in all other previously studied arthropods.

**Fig. 5:**
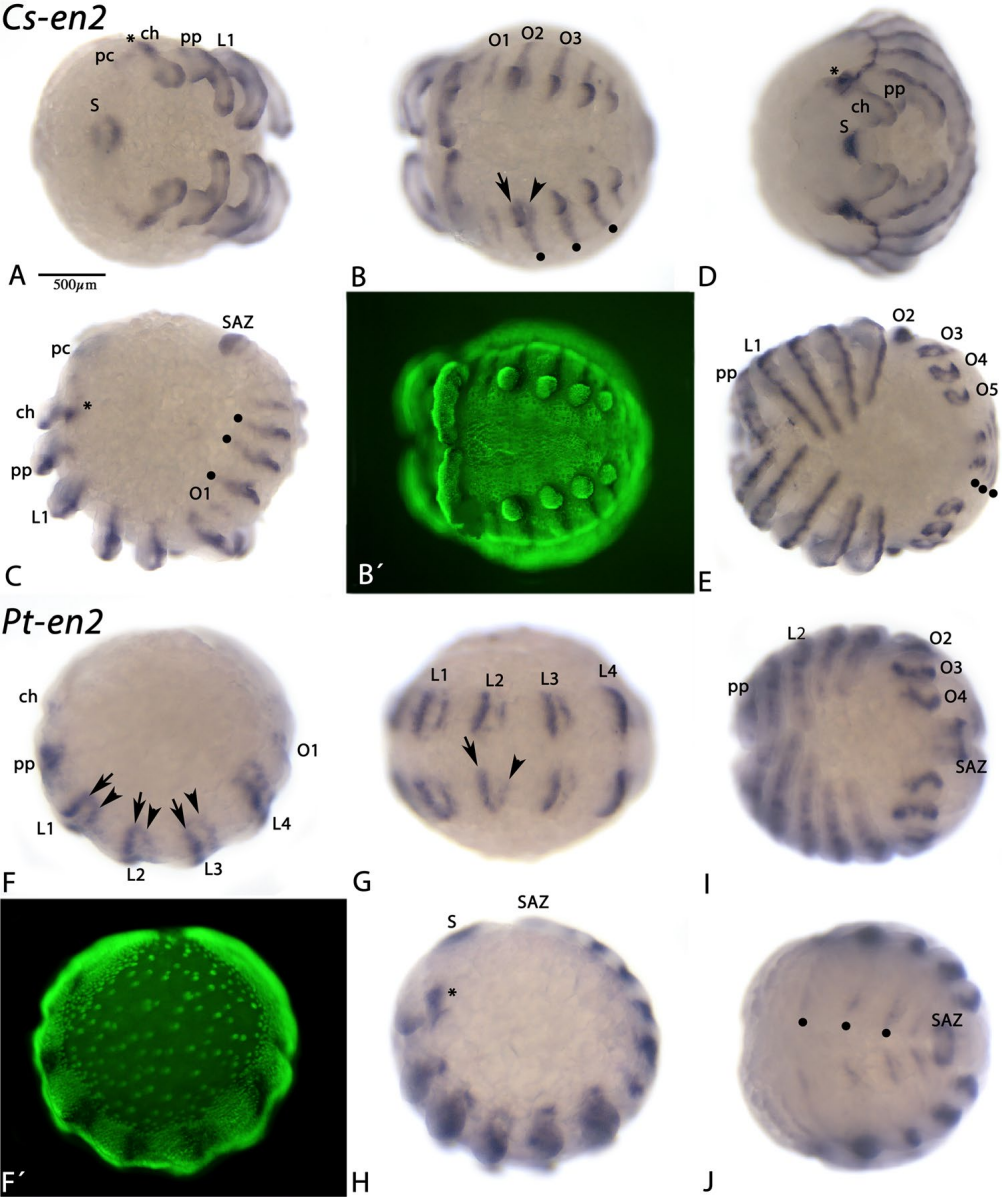
Expression of *Cupiennius* and *Parasteatoda en2*. In all panels, anterior is to the left. Panels A, B, E, G, and I show ventral views. Panel D represents an anterior view. Panels C, F, and H show lateral views. Panel J shows a dorsal view. Panels C´ and F´ represent Sybr-green staining of the embryos shown in panels C and F. In all panels, asterisk mark expression in the ocular region and filled dots mark expression dorsal to the opisthosomal limb buds. Arrows point to the anterior domain of segmental *en2* expression and arrowheads point to the posterior domain of segmental *en2* expression. Abbreviations as in Fig. 2, and pc, precheliceral region; S, stomodaeum; SAZ segment addition zone

**Fig. 6:**
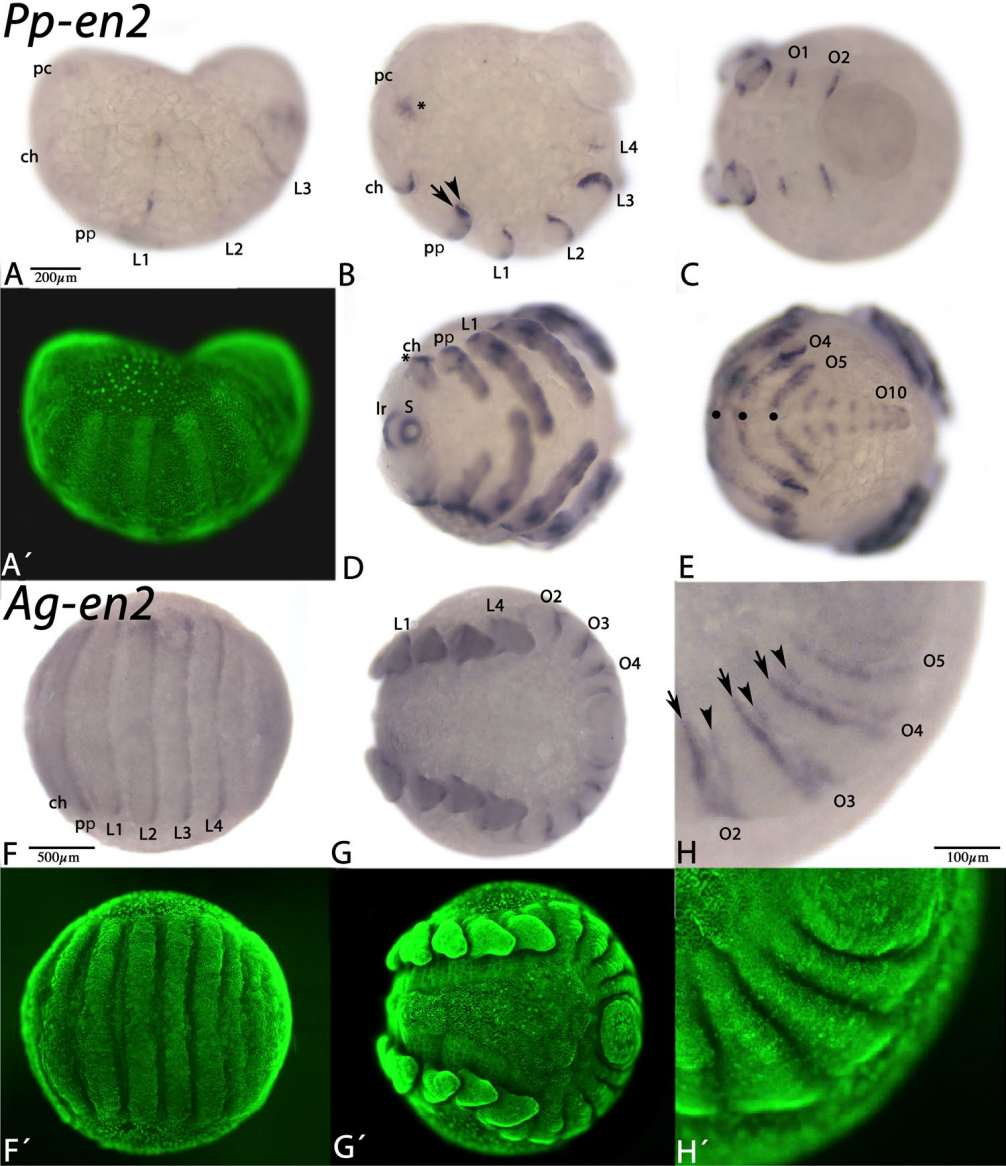
Expression of *Pholcus and Acanthoscurria* en2. In all panels, anterior is to the left. Panels A and B represent lateral views. Panel E shows posterior and dorsal view. The other panels represent ventral views. Panels A´ and F´–H´ represent Sybr-green staining of the embryos shown in panels A and F–H. Asterisks, filled circles, arrows and arrowheads as in Fig. 4. Abbreviations as in Fig. 5, and lr, labrum

**Fig. 7:**
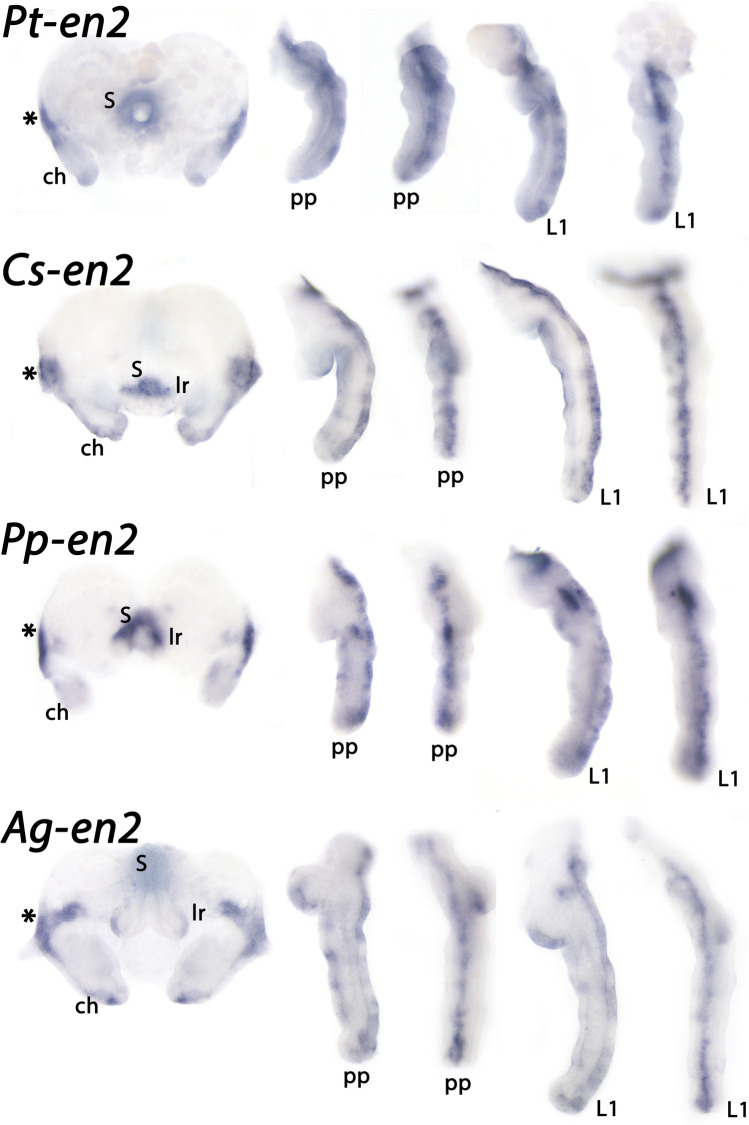
Expression of spider *en2* in the head and the appendages. The anterior head region with the stomodaeum and the chelicerae are shown from ventral. The pedipalps and legs are shown from lateral (left panels) and dorsal (anterior to the right) (right panels). Note expression in the center of the pedipalps and legs. Abbreviations as in Fig. 5

**Fig. 8:**
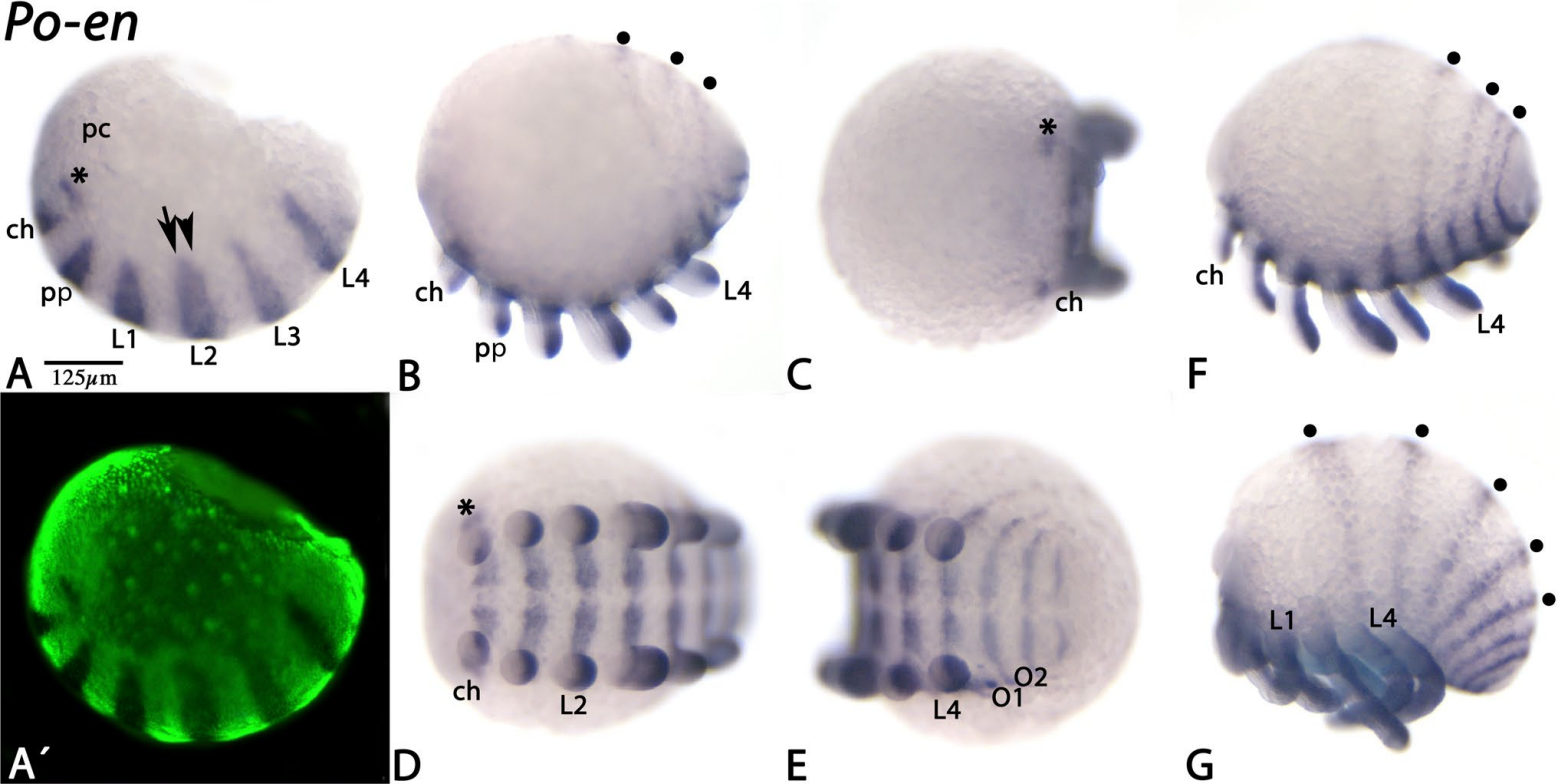
Expression of harvestman *engrailed*. In all panels, anterior is to the left. Lateral views except panel C (anterior view) and panels D and E (ventral views). Panel A´ represents Sybr-green staining of the embryo shown in panel A. Asterisks and filled circles as in Fig. 4. Note that the domain of segmental *en* expression is not split (arrow and arrowhead) and that there is no expression in the stomodaeum. Abbreviations as in Fig. 5

Expression of the arachnopulmonate-specific second *en* paralog (*en2*), however, is different. Although it is expressed in the form of transverse segmental stripes, very similar to the expression of *en1*, the stripes of *en2* are broader and extend further towards posterior spanning the segmental grooves. Consequently, *en2* is expressed anterior (just like *en1*) *and* posterior to these grooves (Figs. 5 and 6). Additionally, spider *en2* genes (but not *en1*) are expressed in the stomodaeum, and expression in the appendages is dorsal and more anterior than that of *en1* (Figs. 5–7). Unlike *en1* genes, however, orthologs of *en2* are not expressed in the ventral nervous system, representing a case of sub-functionalization. Expression of the single harvestman *en* gene is very similar to that of spider *en1*: the segmental stripes are not split, there is no expression of *en* in the stomodaeum, expression in the ventral nervous system is present, and expression in the appendages is posterior (Fig. 8). The pattern of *en2* is thus derived representing a case of neo-functionalization. And since this new pattern is highly conserved in at least spiders (expression data from other arachnopulmonate chelicerates are not available), it must have an important, likely new, role during development. Unfortunately, however, studies concerning the function of *en2* in spiders are lacking. The remaining question is thus what (if any) morphological novelty may be correlated with the function of the second *engrailed* gene?

## Supplementary Information

High resolution (TIF 24265 kb)

High resolution (TIF 8939 kb)

Supplementary file 1 (NEX 48.8 KB)

Supplementary file 1 (MSAPX 19.2 KB)

## Data Availability

All data of this study have been made public.
