# Supplementary figures and images for: Lack of evidence for conserved parasegmental grooves in arthropods
Source: Dev Genes Evol. 2022 Jan 17;232(1):27–37. doi: 10.1007/s00427-022-00684-5 (PMC8918137; doi:10.1007/s00427-022-00684-5)

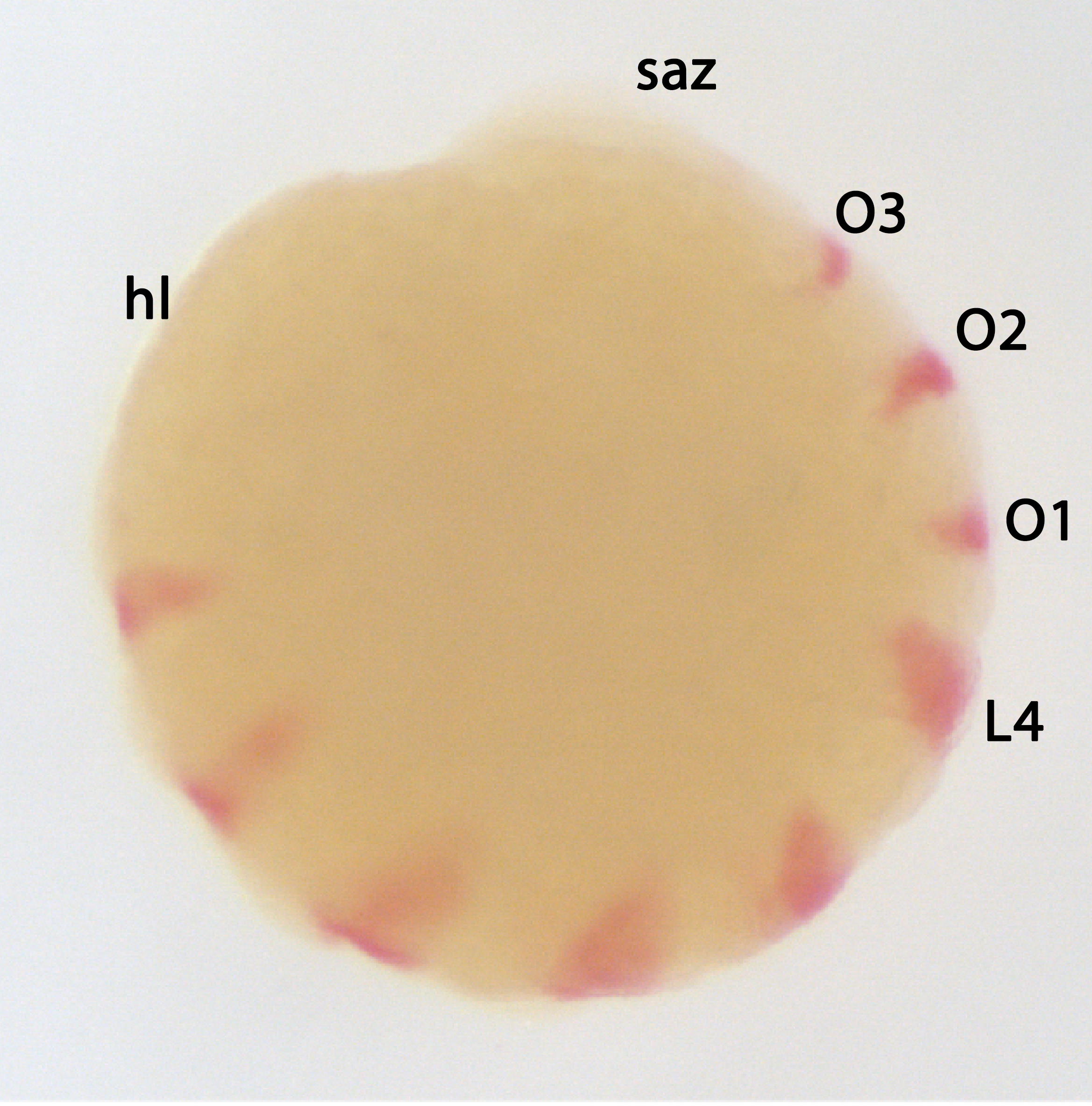

Supplement: Supplementary file 1 [file 427_2022_684_Fig9_ESM.png]

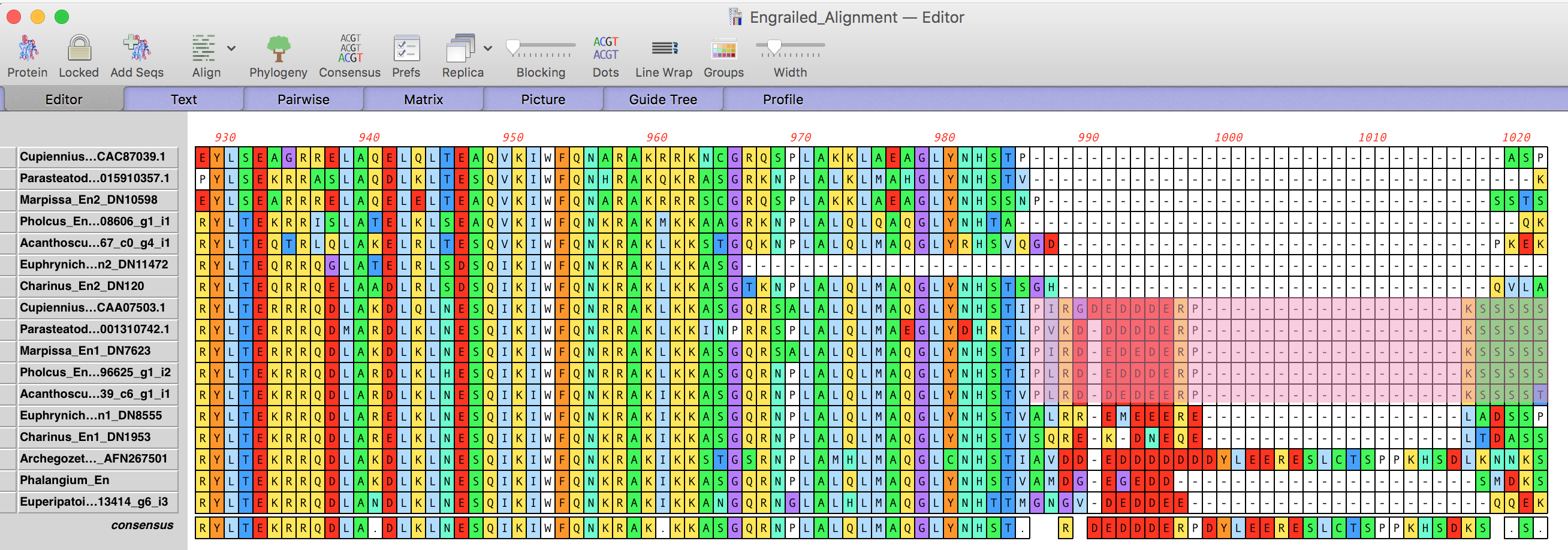

Supplement: Supplementary file 3 [file 427_2022_684_Fig10_ESM.png]

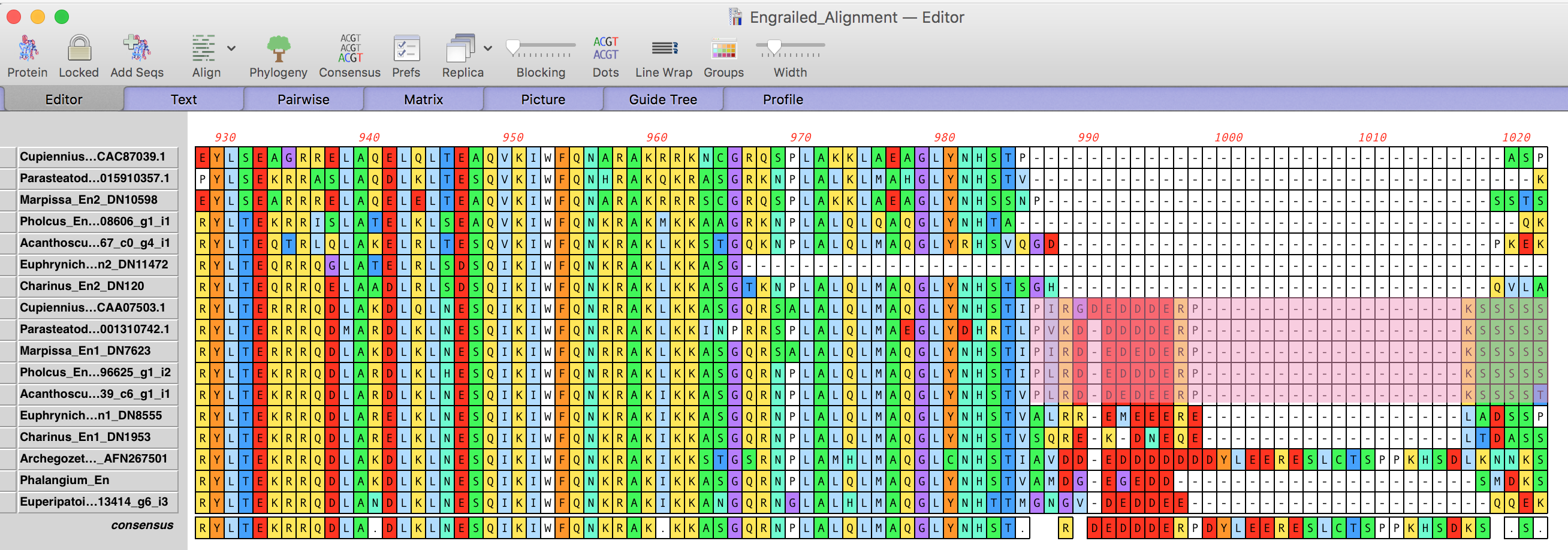

Supplement: Supplementary file 4 — High resolution (TIF 8939 kb) [file 427_2022_684_MOESM2_ESM.tif]
